# Supplementary material for: Polyketide synthase gene clusters in the ericoid mycorrhizal fungus Oidiodendron maius: genome-wide prediction, expression profiling, and functional characterization of knockout mutants
Source: Front Plant Sci. 2026 Sep 16;17:1924430. doi: 10.3389/fpls.2026.1924430 (PMC13623903; doi:10.3389/fpls.2026.1924430)
Supplement: Supplementary file 1 [file SupplementaryFile1.zip › Supplementary Materials/Supplementary Folder 1.zip!/Oidma1_AssemblyScaffolds_Repeatmasked/knownclusterblast/cluster40/jgi.p_Oidma1_133765_mibig_hits.html]

| MIBiG Protein | Description | MIBiG Cluster | MiBiG Product | % ID | % Coverage | BLAST Score | E-value |
| --- | --- | --- | --- | --- | --- | --- | --- |
| EHA28233.1 | hypothetical\_protein | BGC0001143 | Azanigerone\_biosynthetic\_gene\_cluster | 39 | 97.4820143885 | 399 | 7.8e-111 |
| AMY15066.1 | acyl-CoA\_synthetase/ligase | BGC0001339 | Squalestatin\_S1\_biosynthetic\_gene\_cluster | 35 | 95.8633093525 | 306 | 5.3e-83 |
| OAQ83766.1 | phenylacetyl-\_ligase\_protein | BGC0001358 | Leucinostatins\_biosynthetic\_gene\_cluster | 32 | 63.8489208633 | 165 | 2.5e-40 |
| AGY30675.1 | Ann3 | BGC0001298 | 4-Z-annimycin\_biosynthetic\_gene\_cluster | 28 | 89.2086330935 | 153 | 7.5e-37 |
| WP\_010639243.1 | 2-succinylbenzoate--CoA\_ligase | BGC0000958 | Antimycin\_biosynthetic\_gene\_cluster | 28 | 92.2661870504 | 148 | 3.2e-35 |
| AEI98652.1 | CtcI | BGC0000209 | Chlortetracycline\_biosynthetic\_gene\_cluster | 28 | 84.8920863309 | 129 | 1.5e-29 |
| AIL50189.1 | putative\_5-aminolevulinate\_CoA-ligase | BGC0000213 | Colabomycin\_biosynthetic\_gene\_cluster | 27 | 90.8273381295 | 127 | 4.4e-29 |
| ACF35445.1 | MbcAI | BGC0000090 | Macbecin\_biosynthetic\_gene\_cluster | 26 | 84.5323741007 | 115 | 2.3e-25 |
| ADE34493.1 | SsfL2 | BGC0000269 | SF2575\_biosynthetic\_gene\_cluster | 25 | 85.4316546763 | 108 | 2.8e-23 |
| CAO85890.1 | aryl-coA\_ligase | BGC0000110 | Neoaureothin\_biosynthetic\_gene\_cluster | 28 | 63.8489208633 | 107 | 6.2e-23 |
| ABF88003.1 | putative\_long-chain-fatty-acid\_CoA\_ligase | BGC0000871 | VEPE\_/\_AEPE\_/\_TG-1\_biosynthetic\_gene\_cluster | 25 | 80.3956834532 | 101 | 4.4e-21 |
| ABI22133.1 | putative\_non-ribosomal\_peptide\_synthetase | BGC0000422 | Saframycin\_A\_biosynthetic\_gene\_cluster | 24 | 94.4244604317 | 93 | 7.1e-19 |
| CCA65703.1 | anthranilate-CoA\_ligase | BGC0001343 | Aurachin\_biosynthetic\_gene\_cluster | 30 | 43.7050359712 | 91 | 4.6e-18 |
| ACR50797.1 | acyltransferase | BGC0000163 | Tetronasin\_biosynthetic\_gene\_cluster | 23 | 104.856115108 | 88 | 2.3e-17 |
| AEU11005.1 | NpnA | BGC0001029 | Nostophycin\_biosynthetic\_gene\_cluster | 24 | 67.6258992806 | 76 | 8.9e-14 |
| CAA11795.1 | PCZA363.4 | BGC0000322 | Chloroeremomycin\_biosynthetic\_gene\_cluster | 23 | 93.1654676259 | 65 | 2.7e-10 |
| AEW31015.1 | plipastatin\_synthetase | BGC0000407 | Plipastatin\_biosynthetic\_gene\_cluster | 28 | 39.2086330935 | 65 | 2.7e-10 |
| CCB53264.1 | non-ribosomal\_peptide\_synthetase | BGC0001393 | Lugdunin\_biosynthetic\_gene\_cluster | 26 | 40.4676258993 | 62 | 2.3e-09 |
| AKA59447.1 | non-ribosomal\_peptide\_synthetase | BGC0001203 | Clarexpoxcin\_biosynthetic\_gene\_cluster | 24 | 56.2949640288 | 56 | 1.6e-07 |
| CAJ96471.1 | Non-ribosomal\_peptide\_synthetase | BGC0000330 | Cupriachelin\_biosynthetic\_gene\_cluster | 26 | 29.4964028777 | 55 | 2.8e-07 |
